# Virtual Reality for Non-Ordinary Consciousness

**DOI:** 10.3389/frobt.2018.00007

**Published:** 2018-02-07

**Authors:** Gabriel Axel Montes

**Affiliations:** ^1^University of Newcastle, Newcastle, NSW, Australia; ^2^Priority Research Centre for Stroke and Brain Injury, Hunter Medical Research Institute, Newcastle, NSW, Australia; ^3^Research in Bias Node, Charles Perkins Centre, University of Sydney, Sydney, NSW, Australia

**Keywords:** consciousness, virtual reality, meditation, neurological disorders, religion and science, yoga, shamanism, embodied cognition

Virtual reality (VR) technology is currently seeing a surge of interest in industry, academia, and the public. Myriad developments are already underway, aiming to bring the technology directly to users in ways that offer access to rich virtual multisensory experiences. The immersivity that VR offers is enabled by the brain’s constraints on processing bodily self-consciousness (BSC), which anchors self-identity and -location to the physical body. Current developments are focused on leveraging the face-value constraints of BSC to craft immersive VR experiences that are plausible to the human user; i.e., most VR applications today take advantage of BSC as a “trick” or illusion that the body plays on the mind. However, the malleability of BSC can be more powerfully approached as an asset for enhancing the repertoire of body representations and plasticity available to everyday human experience. By manipulating the boundaries of self-local experience, VR can be used as a tool for the cultivation of non-ordinary consciousness (NOC). Such an approach would have the potential to equip society with novel pathways for studying the farther reaches of consciousness and providing opportunities for access to enhanced conscious experiences in everyday life, with far-reaching philosophical and ethical implications.

## Bodily Self-Consciousness

A growing body of work in cognitive neuroscience and philosophy of mind has characterized the multisensory mechanisms governing the integration of bodily signals on which VR relies, or what is called BSC (Blanke et al., 2015). Beginning in neurological patients, this work has extended into healthy subjects and received much attention with bodily illusion paradigms, such as the rubber-hand illusion, enfacement illusion, and the full-body illusion, in which a study subject experiences the subject sense that a hand, face, or other body, respectively, is their own. The full-body illusion was developed using VR, and the rubber hand and enfacement illusions, while developed without using VR, were eventually applied in VR. These paradigms have revealed that such illusory experiences can be achieved through the spatiotemporal manipulation of visuo-tactile, and sometimes vestibular and auditory, inputs in relation to body parts, either directly or within the behaviorally and neurophysiologically defined potential space—the peripersonal space (PPS)—immediately surrounding the stimulated body part. Empirical data suggest two major brain networks underlying BSC: a frontoparietal one (intraparietal sulcus and premotor cortex; IPS and PMC, respectively) for processing signals related to circumscribed body parts, e.g., hand and face, and a temporoparietal one (supramarginal gyrus, insula, superior temporal gyrus) for processing signals for trunk-based self-identification and self-location. In addition, it has been demonstrated that vestibular processing contributes significantly to anchoring self-location with the physical body (Pfeiffer et al., 2013).

Four principal constraints on multisensory integration have been proposed and evidenced to modulate BSC: (1) proprioception, (2) body-related visual information, (3) PPS, and (4) embodiment. These constraints determine whether or not a change in BSC takes place, fulfilling the criteria for the aforementioned bodily illusions to occur. The result is a subjective (and objectively measurable) sense of ownership over the virtual/prosthetic body part, e.g., the rubber hand (Botvinick and Cohen, 1998; Apps and Tsakiris, 2014), self-identification with the full body, and self-location (Blanke and Metzinger, 2009; Blanke, 2012). The integration of bodily ownership with motor signals yields the sense of agency, or volition over one’s actions (Tsakiris et al., 2007; Ma and Hommel, 2015; Trzepacz et al., 2015; Haggard, 2017). The BSC constraints are modulated by the confluence of hierarchical bottom-up and top-down streams of information in the nervous system, which have been formalized in Bayesian models of predictive coding (Clark, 2013; Samad et al., 2015) and the free-energy principle (Friston, 2010; Apps and Tsakiris, 2014; Pezzulo et al., 2015; Donnarumma et al., 2017). In the rubber-hand illusion, for example, bottom-up unisensory inputs (touch on the real hand and vision of the rubber hand) are integrated in multisensory areas according to predictions based on prior experiences (Hohwy and Paton, 2010). The multisensory incongruity produces a mismatch between the sensory and predictive streams of information, leading to an attempt at its resolution (prediction error minimization) by weighing the proprioceptive input higher than the visual incongruence, thus producing embodiment of the rubber hand. These models help explain why the virtual environments can elicit such a convincing sense of ownership and agency over illusory bodies and achieve a feeling of presence.

While the behavioral, neural, and theoretical evidence supporting BSC are well defined, the research field currently treats the variability and plasticity of BSC as a “trick” or mere illusion that besets a static physical body, which the brain then attempts to correct. The concept of an illusion is apt as a convention for referencing the deviational phenomena; however, a strict adherence to this notion betrays the implication of the evidence that self-localization is phenomenologically associated with the physical body *because* of the very mechanisms of multisensory information processing, which are biased in favor of heavily weighted-predictive priors on the physical body (trunk BSC especially) (Blanke, 2012; Blanke et al., 2015), rather than due to the body being an *a priori* ontological locus of selfhood—the “self-localization fallacy.” Self-location coincides with the physical body because it is, under normal conditions, the nexus where the various sensory streams converge and with respect to which they are globally integrated. The physical body thereby becomes a statistically evergreen Bayesian prior that strongly conditions perception to perceive consciousness as being anchored to, or localized in, it. This view remains commensurate with the starting point that consciousness is produced by the brain while concurrently remaining neutral regarding an ontologically fixed locality (or non-thereof) of selfhood.

## Non-Ordinary Consciousness

Resetting the self-localization assumption and leveraging the insights of BSC-related scientific evidence offer a foundation for manipulating BSC for hitherto-unexplored ends, particularly through taking advantage of VR. Whereas in the case of neurological or psychiatric patients who experience pathological BSC distortions of self-identification and -localization, the goal might be to promote a healthier and more physically bound BSC (Blanke, 2012), in healthy subjects with neurotypical BSC where there is more room to manipulate and expand the ordinary boundaries of physically bound BSC. In support of this idea is the fact that many practitioners of meditation, yoga, and other methods of NOC report that such practices result in a decreased identification with their physical body and an increased sense of overall well-being (Vago and David, 2012; Tang et al., 2015; Montes, 2017). In line with these reports, the cross-cultural purported purpose of many of the practices of the world’s wisdom and shamanic traditions (e.g., Buddhism, contemplative Christianity, Sufism, Taoism, Hinduism, etc.) is to reduce overidentification with the physical body and ego-self, as a means of ameliorating mental-emotional suffering. Many of the NOC practices associated with these traditions directly operate on BSC, such as out-of-body experiences, lucid dreaming, cultivating alternate body schemas and models, qi gong, hypnagogic states, and heightened interoceptive awareness (which crucially involves the insula and a brain region that also regulates self-identification and self-location) (Aspell et al., 2013; Ronchi et al., 2015), among myriad others. The methods of NOC thereby offer a treasury of techniques by which to entrain non-ordinary BSC for both consciousness-related scientific research and education. Opening this line of investigation honors the underappreciated insight of BSC research that the sense of self is experienced as bound to the physical body because of the mechanisms of embodied multisensory integration and hierarchical-predictive weighting, not because it is inherently bound *per se* to the body.

While for many scientists, NOC methods may seem out of reach or experimentally intractable, recent research has made sizeable progress in parsing them into core cognitive domains that may be studied in the laboratory and are easily manipulated using VR (Vago and David, 2012; Tang et al., 2015; Montes, 2017). NOC practices typically enact a set of constructs in cognitive neuroscience, which are supported by phenomenological, behavioral, and neuroscientific evidence: attention, intention, expansion/evocation, refinement, engagement, and evaluation. While some models prefer more generalizable constructs (e.g., attention, self-regulation, self-awareness) (Vago and David, 2012; Tang et al., 2015), maintaining both specificity and adaptability helps to strike a balance that more precisely anchors NOC methods in both cognitive neuroscience and their rich phenomenology (Montes, 2017). Furthermore, a framework/model may possess broader and deeper applicability if it accounts for computational principles of cognition, such as predictive coding. Because of the phenomenological nature of NOC methods, it is also advantageous if a model can be used to not only measure, but crucially, to entrain and enact those methods. This fosters a neurophenomenological fluidity and flexibility conducive to both research on NOC and education on conducting its practices by cultivators.

Informed by the constraints of BSC and the insights of predictive coding, VR is a powerful means of entraining NOC methods for the down-weighting of self-localization priors and expanding into and integrating alternate body schemas. Particular spatiotemporal combinations of multisensory—and even brain—stimulation can be explored and systematized to reliably and progressively induce the ownership of and agency over virtual or robotic bodies. The virtual bodies may have different scale, form, consistency, number, interactivity parameters, etc. to achieve desired effects and would be embodied by the user in progressively greater orders of magnitude so as to diversify the user’s body priors and entrain novel ones (Hohwy and Paton, 2010). Newly conditioned body schemas and NOC can be integrated with the physical body so that the individual may maintain a healthy relationship with the four-dimensional world of space-time while cultivating and assimilating new body priors. While there is much research remaining to be done regarding NOC, the time is ripe for both academic and industry efforts to explore this synergy between VR and NOC.

## Societal Implications

As the use of VR technology grows increasingly widespread, the philosophical, ethical, and societal implications will likewise continue to grow. The attenuation of physical body Bayesian priors that could come with conditioning to alternate worlds and embodiment dynamics (especially in children growing up with extensive VR use) may result in an experience of a greater pre-reflective readiness to take ownership over virtual bodies. Such experiences may give credence to the self-localization fallacy and raise new philosophical questions about the nature of consciousness and embodiment. Ethically, it will be important to recognize the potency of VR-enabled NOC and to remain mindful about overly relying on VR beyond it, providing “primer” experiences that can then be cultivated without VR.

The potential for a huge societal impact will be mainly in the use of VR for education on NOC; access to NOC will be democratized and more readily available to non-practitioners. Scientific research will also be able to harness the new technology to conduct previously inaccessible experimental paradigms. Virtual embodiment of alternate body schemas will enable a spectrum of BSC exploration and facilitate NOC experiences in potentially safe and systematic ways. User data collection and analytics and computational models of BSC and NOC can fuel artificial intelligence (AI) engines that guide users through BSC experiences in VR according to their strengths and weaknesses. While it remains to be seen if NOC VR will make a significant contribution to the amelioration of human suffering as maintained by the practices/traditions that might inform or inspire NOC VR methods, the immersive embodiment afforded by VR coupled with the repository of available NOC methods suggests promising potential in this area.

In addition to democratizing NOC for clinically healthy individuals, NOC VR will also be able to serve clinical neurological populations. Existing research already reveals impaired BSC in neurological patients, including disordered self-localization (Heydrich and Blanke, 2013). Non-ordinary BSC will deepen the scientific understanding of BSC and open new doors for offering BSC therapies and facilitate the embodiment of robotic bodies. Furthermore, in a future society where there may be imprecise priors for embodiment and BSC due to the prevalence of immersion in VR, NOC-informed VR therapies for the normalization of BSC may become an industry, with room for technological and medical innovation. Such scenarios may necessitate diverse training in the cognitive neuroscience of BSC, clinical manifestations of BSC disorders, mechanisms of VR, and experience with NOC that is grounded in both empirical science and phenomenology.

Taken together, VR and NOC are poised to form a mutually beneficial alliance in service of BSC treatment and enhancement. Neurological disability could make use of VR-assisted BSC therapy, and healthy individuals may harness the multisensory stimulation afforded by VR to embody alternate body schemas as a means of dilating the bounds of accessible conscious experience. With the assistance of AI, it will be possible to create experiences and programs tailored to individual needs and wishes. Importantly for researchers in academia and industry alike, NOC VR affords opportunities for generating experience-driven hypotheses and experimental paradigms for consciousness research. Balancing the exploration and cultivation of both physically embodied and non-ordinary BSC, society is set to reap the rewards of the intrepid exploration of BSC potentiated by VR.

## Author Contributions

GAM conceived, drafted, and revised this work in its entirety.

## Conflict of Interest Statement

The author declares that the research was conducted in the absence of any commercial or financial relationships that could be construed as a potential conflict of interest.

## References

[B1] Apps M. A. J. Tsakiris M. (2014). The free-energy self: a predictive coding account of self-recognition. Neurosci. Biobehav. Rev. 41, 85–97. 10.1016/j.neubiorev.2013.01.029 23416066 PMC3848896

[B2] Aspell J. E. Heydrich L. Marillier G. Lavanchy T. Herbelin B. Blanke O. (2013). Turning body and self inside out. Psychol. Sci. 24, 2445–2453. 10.1177/0956797613498395 24104506

[B3] Blanke O. (2012). Multisensory brain mechanisms of bodily self-consciousness. Nat. Rev. Neurosci. 13, 556–571. 10.1038/nrn3292 22805909

[B4] Blanke O. Metzinger T. (2009). Full-body illusions and minimal phenomenal selfhood. Trends Cogn. Sci. 13, 7–13. 10.1016/j.tics.2008.10.003 19058991

[B5] Blanke O. Slater M. Serino A. (2015). Behavioral, neural, and computational principles of bodily self-consciousness. Neuron 88, 145–166. 10.1016/j.neuron.2015.09.029 26447578

[B6] Botvinick M. Cohen J. (1998). Rubber hands ‘feel’ touch that eyes see. Nature 391, 756 10.1038/35784 9486643

[B7] Clark A. (2013). Whatever next? Predictive brains, situated agents, and the future of cognitive science. Behav. Brain Sci. 36, 181–204. 10.1017/S0140525X12000477 23663408

[B8] Donnarumma F. Costantini M. Ambrosini E. Friston K. Pezzulo G. (2017). Action perception as hypothesis testing. Cortex 89, 45–60. 10.1016/j.cortex.2017.01.016 28226255 PMC5383736

[B9] Friston K. (2010). The free-energy principle: a unified brain theory? Nat. Rev. Neurosci. 11, 127–138. 10.1038/nrn2787 20068583

[B10] Haggard P. (2017). Sense of agency in the human brain. Nat. Rev. Neurosci. 18, 196–207. 10.1038/nrn.2017.14 28251993

[B11] Heydrich L. Blanke O. (2013). Distinct illusory own-body perceptions caused by damage to posterior insula and extrastriate cortex. Brain 136, 790–803. 10.1093/brain/aws364 23423672

[B12] Hohwy J. Paton B. (2010). Explaining away the body: experiences of supernaturally caused touch and touch on non-hand objects within the rubber hand illusion. PLoS ONE 5:e9416. 10.1371/journal.pone.0009416 20195378 PMC2827559

[B13] Ma K. Hommel B. (2015). The role of agency for perceived ownership in the virtual hand illusion. Conscious. Cogn. 36, 277–288. 10.1016/j.concog.2015.07.008 26196450

[B14] Montes G. A. (2017). “Non-ordinary consciousness for artificial intelligence,” in Biomimetic and Biohybrid Systems: 6th International Conference, Living Machines 2017, Stanford, CA, USA, July 26–28, 2017, Proceedings, eds Mangan M. Cutkosky M. Mura A. Verschure P. F. M. J. Prescott T. Lepora N. (Cham: Springer International Publishing), 348–362.

[B15] Pezzulo G. Rigoli F. Friston K. (2015). Active inference, homeostatic regulation and adaptive behavioural control. Prog. Neurobiol. 134, 17–35. 10.1016/j.pneurobio.2015.09.001 26365173 PMC4779150

[B16] Pfeiffer C. Lopez C. Schmutz V. Duenas J. A. Martuzzi R. Blanke O. (2013). Multisensory origin of the subjective first-person perspective: visual, tactile, and vestibular mechanisms. PLoS ONE 8:e61751. 10.1371/journal.pone.0061751 23630611 PMC3632612

[B17] Ronchi R. Bello-Ruiz J. Lukowska M. Herbelin B. Cabrilo I. Schaller K. (2015). Right insular damage decreases heartbeat awareness and alters cardio-visual effects on bodily self-consciousness. Neuropsychologia 70, 11–20. 10.1016/j.neuropsychologia.2015.02.010 25676677

[B18] Samad M. Chung A. J. Shams L. (2015). Perception of body ownership is driven by Bayesian Sensory Inference. PLoS ONE 10:e0117178. 10.1371/journal.pone.0117178 25658822 PMC4320053

[B19] Tang Y.-Y. Holzel B. K. Posner M. I. (2015). The neuroscience of mindfulness meditation. Nat. Rev. Neurosci. 16, 213–225. 10.1038/nrn3916 25783612

[B20] Trzepacz P. T. Hochstetler H. Wang S. Walker B. Saykin A. J. For the Alzheimer’s Disease Neuroimaging, I. (2015). Relationship between the Montreal Cognitive Assessment and Mini-mental State Examination for assessment of mild cognitive impairment in older adults. BMC Geriatr. 15:107. 10.1186/s12877-015-0103-3 26346644 PMC4562190

[B21] Tsakiris M. Schutz-Bosbach S. Gallagher S. (2007). On agency and body-ownership: phenomenological and neurocognitive reflections. Conscious. Cogn. 16, 645–660. 10.1016/j.concog.2007.05.012 17616469

[B22] Vago D. R. David S. A. (2012). Self-awareness, self-regulation, and self-transcendence (S-ART): a framework for understanding the neurobiological mechanisms of mindfulness. Front. Hum. Neurosci. 6:296. 10.3389/fnhum.2012.00296 23112770 PMC3480633

